# The flower does not open in the city: evolution of plant reproductive traits of *Portulaca oleracea* in urban populations

**DOI:** 10.1093/aob/mcae105

**Published:** 2024-08-01

**Authors:** Tomohiro Fujita, Naoe Tsuda, Dai Koide, Yuya Fukano, Tomomi Inoue

**Affiliations:** National Institute for Environmental Studies, Ibaraki, Japan; National Institute for Environmental Studies, Ibaraki, Japan; National Institute for Environmental Studies, Ibaraki, Japan; Graduate School of Horticulture, Chiba University, Chiba, Japan; National Institute for Environmental Studies, Ibaraki, Japan

**Keywords:** *Portulaca*, urban, cleistogamy, pollinator loss, heat stress, drought, phenology, seed weight, adaptation, evolution

## Abstract

**Background and Aims:**

The impact of urbanization on plant evolution, particularly the evolution of reproductive traits, remains largely unknown. In this study, we aimed to investigate the consequences of urbanization on the reproductive traits of *Portulaca oleracea* in the Kantō region of Japan. *Portulaca oleracea* has a unique cleistogamous reproductive system, which consists of genetically determined chasmogamous (open, CH) and cleistogamous (closed, CL) plants.

**Methods:**

We collected seeds of *P*. *oleracea* from ten populations in rural areas and ten populations in urban areas. In a common garden experiment, we recorded the type of flowers (CH or CL), reproductive phenology and seed production.

**Key Results:**

All individuals produced either CH or CL flowers, allowing us to classify them as either CH or CL plants. We observed a significant difference in the prevalence of CH and CL plants between rural and urban populations: the number of CH plants was generally low and was particularly low among urban individuals. Compared to CH plants, CL plants showed earlier phenology and produced heavier seeds, which is consistent with stress avoidance in response to heat and drought stress conditions in urban areas.

**Conclusions:**

Our findings suggest that urbanization may drive an evolutionary change in the cleistogamous reproductive system of *P*. *oleracea*. CL plants with earlier phenology and larger seeds might be better adapted to urban environments, where they are subjected to harsh heat and drought stress.

## INTRODUCTION

Global urbanization is rapidly changing the face of the Earth. Approximately 3 % of the land surface has been covered by urban development (Grimm *et al.*, 2008), and urban areas are continuing to expand throughout the world (United Nations, 2015). Urbanization causes pronounced changes in the biotic and abiotic environments: changes in abiotic factors include an increase in temperature and pollution, and changes in biotic factors include the reduced abundance of pollinators and herbivores and altered species interactions compared to those in non-urban areas (McKinney, 2002; Pickett *et al.*, 2011; Aronson *et al.*, 2016; Miles *et al.*, 2019; Wenzel *et al.*, 2020; Liang *et al.*, 2023). Such artificially altered urban habitats exert new environmental pressures that may facilitate biological evolution (McDonnell and Hahs, 2015). Although it is known that urbanization can have large ecological effects on plant and animal communities (Hahs *et al.*, 2009), the evolutionary consequences of urbanization are still poorly understood (Johnson *et al.*, 2015).

Urbanization may increase phenotypic differences between plants from populations in urban and non-urban habitats (Cheptou *et al.*, 2008; Brys and Jacquemyn, 2012; Johnson *et al.*, 2015; Thompson *et al.*, 2016; Johnson and Munshi-South, 2017; Yakub and Tiffin, 2017; Fukano *et al.*, 2020, 2023; Santangelo *et al.*, 2020; Santangelo *et al.*, 2022*a*, 2022*b*; Ishiguro *et al.*, 2023; Taichi and Ushimaru, 2023). For example, *Lepidium virginicum* exhibits notable phenotypic differences, such as in reproductive phenology, between populations in urban and adjacent rural areas in the northern United States (Yakub and Tiffin, 2017). Cheptou *et al*. (2008) analysed seed dispersal traits in *Crepis sancta* in France and found that plants in urban environments have evolved to produce a higher proportion of non-dispersing seeds than plants in rural settings. Santangelo *et al*. (2022*a*) gathered data on *Trifolium repens* populations from 160 cities globally and discovered that the synthesis of anti-herbivore defence chemicals is correlated with distance from urban centres in many cities. Although these studies have provided important insights into the microevolutionary processes in urban plants, the evolutionary consequences of urbanization on plant reproductive traits are still unknown (Brys and Jacquemyn, 2012; Ushimaru *et al.*, 2014).

Cleistogamy is a mixed reproductive strategy encompassing the production of both perpetually closed, obligately self-pollinated flowers (cleistogamous, CL) and open, insect-pollinated flowers (chasmogamous, CH), which are potentially capable of outcrossing (Culley and Klooster, 2007; Stojanova *et al.*, 2020). Corolla and stamen size and/or stamen number are smaller in CL flowers than in CH flowers, and thus CL flowers are less costly to produce than CH flowers (Culley and Klooster, 2007). Cross-pollinated CH flowers generate genetically diverse offspring, maintaining or enhancing genetic diversity (Culley and Klooster, 2007). The occurrence of CH and CL flowers is influenced by various abiotic and biotic factors (Culley and Grubb, 2003; Culley and Klooster, 2007; Sternberger *et al.*, 2020; Carbone *et al.*, 2021). Limited abiotic resources such as soil moisture typically enhance cleistogamy, as CL flowers offer a superior fitness-to-cost ratio, optimizing reproductive success (Culley and Klooster, 2007; Sternberger *et al.*, 2020). CL flowers also ensure seed production through self-pollination even in the absence or scarcity of pollinators (Culley and Klooster, 2007; Panique and Caruso, 2020). Urbanization affects the factors that can influence the production of CH and CL flowers (Johnson *et al.*, 2015); therefore, it may impact the evolution of cleistogamy, but empirical research on this topic is lacking.

*Portulaca oleracea* is an ideal species for assessing the evolutionary consequences of urbanization on the cleistogamous reproductive system. This summer-annual species is distributed globally and thrives in agricultural and urban environments, such as fields, roadsides and railway tracks. *Portulaca oleracea* is known for its unique cleistogamous reproductive system. Unlike in many other cleistogamous species (Culley and Grubb, 2003; Culley and Klooster, 2007; Sternberger *et al.*, 2020; Carbone *et al.*, 2021), the production of CH or CL flowers in *P*. *oleracea* is suggested to be a genetically determined characteristic (Furukawa *et al.*, 2020). Each *P*. *oleracea* individual produces either CH or CL flowers, and flower production does not change plastically (Furukawa *et al.*, 2020).

In this study, we hypothesized that urbanization influences the evolution of reproductive traits in *P*. *oleracea*. To examine this hypothesis, we conducted a common garden experiment using seeds of *P*. *oleracea* collected from ten urban and ten rural habitats near Tokyo, Japan. By growing all of the samples in the same environment, we were able to examine the genetic differences in the occurrence of CH and CL plants, reproductive phenology and seed production. We also examined the differences in reproductive phenology and seed production traits of the same type (e.g. CL) between urban and rural populations. We addressed three key questions: (1) Does the relative frequency of CH and CL plants differ between urban and rural habitats? (2) Do genetic variations exist in reproductive phenology and seed production traits between CH and CL plants? (3) Are there genetic differences in reproductive phenology and seed production traits within the same type?

## MATERIALS AND METHODS

### Study species

**Fig. 1.**
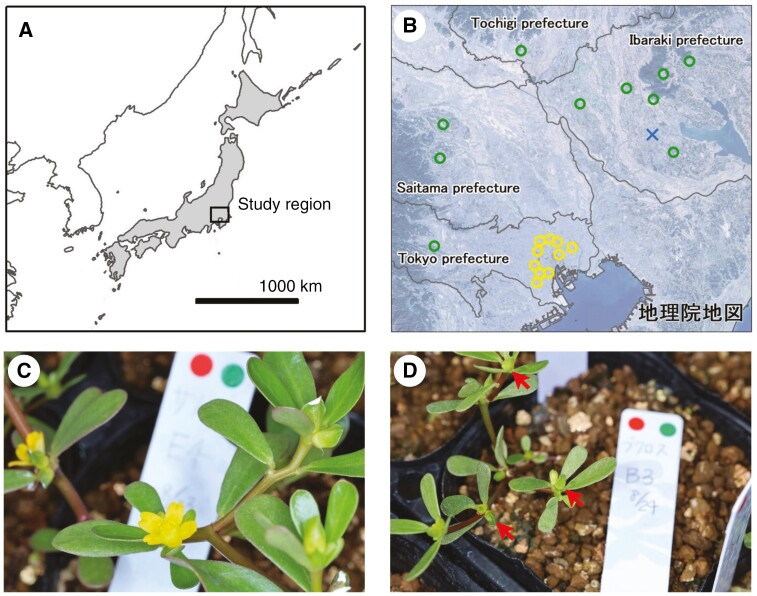
(A) Geographic map of East Asia. The Japanese Archipelago is in grey; the study region is enclosed in a rectangle. (B) Map of the locations of the seed sampling points and the common garden experiment. Urban populations are represented by yellow circles, rural populations by green circles and the experiment location by a blue cross mark. (C) Images of chasmogamous flowers of *Portulaca oleracea*. (D) Images of cleistogamous flowers of *P*. *oleracea*, which are indicated by arrows.

*Portulaca oleracea* (Portulacaceae) is distributed in temperate and tropical zones, including Japan. The main stems (15–30 cm) grow along the ground. The CH flowers are 5–10 mm in diameter when open and are yellow (Fig. 1). The CH flowers open for a few hours in the morning and never open again once they close, allowing both outcrossing and self-fertilization, whereas the CL flowers have petals but are perpetually closed, allowing only for self-fertilization (Fig. 1) (Furukawa *et al*., 2020).

### Field sampling and environmental variables

*Portulaca oleracea* seeds were collected in 2021 from 20 populations: ten from urban and ten from rural habitats near Tokyo, Japan (Supplementary Data Table S1 and Fig. 1). The urban populations were from the highly urbanized Tokyo area, whereas the rural populations were mainly from cultivated lands such as paddy fields and farmlands. In each population, we collected seeds from two to nine individuals, each growing at least 3 m apart. The seeds were stored under ambient room conditions.

Accurate identification of CH vs. CL plants in a natural population is difficult because even CH plants rarely open flowers, especially on cloudy days (Furukawa *et al.*, 2020). Additionally, the flowering periods of *P*. *oleracea* are limited, and we did not observe any flowering individuals during seed sampling. To evaluate cleistogamy in *P*. *oleracea*, we had to use the common garden approach (see below), rather than field observation.

To identify the factors influencing the occurrence of CH and CL plants, we obtained data on the following environmental variables: land surface temperature (LST), elevation estimated using a digital elevation model and potential evapotranspiration (PET) around the seed sampling locations (Supplementary Data Table S2). These variables were averaged over a 100-m radius surrounding the collection points. Other values such as Normalized Difference Vegetation Index (NDVI), annual aridity and global artificial impervious surface were collected, but they were highly correlated and not used in the analysis.

### F2 generation preparation

To separate evolutionary changes from maternal effects (Franks and Weis, 2008), we cultivated a refresher generation in April 2022 in a glasshouse. Seeds were sown on moist filter paper in Petri dishes. After cotyledon expansion, three individuals from each maternal line were randomly selected and transplanted into plastic pots (9 cm in diameter; 0.36 L in volume) containing a mix of cultivated soil (Akadama soil and vermiculite in a 1:1, v/v, ratio). These plants were grown under natural light in the glasshouse with a controlled temperature of 25 °C. Chemical fertilizer (0.1 g per pot, NPK 8-8-5) was applied, and the pots were watered every 2–3 d. All individuals were bagged to ensure the production of F2 seeds by self-pollination. From these individuals, we harvested ripe seeds for use in the common garden experiment.

### Common garden experiment

In August 2022, a common garden experiment was conducted using F2 generation seeds in the same glasshouse. The F2 seeds were sown and the plants were grown as described above. Three replicates per maternal line were used, totalling 291 individuals (135 individuals from 45 rural maternal lines and 156 individuals from 52 urban maternal lines). In each population, one to nine maternal lines were selected (Supplementary Data Table S1). The maternal line count was lower in the F2 than in the F1 generation because of insufficient seed production from some mother plants; such maternal lines were excluded. F2 individuals with at least one bud (288 in total) were included in the analysis.

The dates of bolting and the appearance of the first ripe fruit were monitored for 85 d post-planting. Bolting was defined as a bud exceeding 1 mm in length and ripening as the opening of the upper half of the pyxidium, indicating seed maturity. Stem length, indicative of plant size, was measured on the day of emergence of the first flower bud. The numbers of opened CH flowers were recorded daily. All individuals produced exclusively either CH or CL flowers, allowing us to classify them as CH or CL plants. The number of mature fruits was recorded at 1- to 3-d intervals throughout the observation period, with three to eight fruits collected per individual. Seeds were counted, their total weight per fruit was measured and the average seed weight was calculated. The total number of seeds produced per individual was estimated as the average number of seeds per fruit multiplied by the number of fruits.

### Statistical analysis

Statistical analysis was conducted in R 4.2.2 (R Core Team, 2022). The chi-square test was used to examine the difference in the prevalence of CL and CH plants between rural and urban populations. A generalized linear mixed model (GLMM) with a binomial error distribution and logit link function was used to investigate the effect of environmental variables at the seed collection sites on the prevalence of CH and CL plants in the populations. The response variable was the reproductive system of each individual (coded as 1 for CH and 0 for CL plants). Fixed effects included habitat type (rural or urban) and non-correlated environmental variables (LST, PET and elevation), standardized to a mean of 0 and variance of 1. The random effect was attributed to the individual plant. For each maternal line, three individuals were cultivated and their seed weight, stem length, growth period length, number of fruits and number of seeds were measured. Average values for each maternal line were used in the analysis. These traits were compared between habitats or reproductive system (CH or CL) using linear mixed modelling assuming a Gaussian distribution. Average values of these traits were used as response variables, with habitat or reproductive system (CH or CL) as fixed effects and population as a random effect. The analyses used the ‘lmer’ function in the ‘lme4’ package.

## RESULTS

### Occurrence of CH and CL plants

**Fig. 2.**
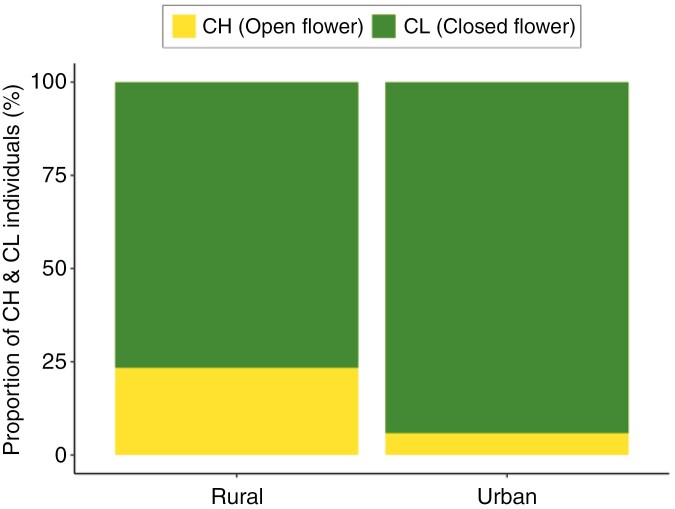
Proportion of chasmogamous (CH) and cleistogamous (CL) *Portulaca oleracea* individuals in the rural and urban populations (*n* = 288 in total). A common garden experiment was conducted with F2-generation seeds. Significance of the difference between the populations was assessed using a chi-square test.

In a common garden experiment, the prevalence of CL and CH plants differed significantly between rural and urban populations (χ^2^ = 16.9, *P* < 0.01, Fig. 2). Of the 288 individuals examined, 248 were identified as CL plants and 40 as CH plants. The proportion of CH plants was particularly low (5.3 %) in the urban population, whereas it was 23.3 % in the rural population.

We used a GLMM analysis to explore the influence of sampling location on the occurrence of CH and CL plants. In addition to habitat type (urban or rural), we incorporated three environmental factors (LST, PET, elevation) as fixed effects. Of the four fixed effects linked to the sampling sites, only LST significantly influenced the presence of CH plants: the odds of CH plant occurrence decreased with an increase in LST at the original collection site (Table 1).

**Table 1.** Analysis of factors affecting the frequency of CH and CL individuals

	Estimate	s.e.	*P*-value
(Intercept)	−0.8436	12.7964	0.947
Habitat type	−37.5845	29.7806	0.207
PET	22.5003	12.599	0.074
Elevation	−0.2757	3.8229	0.942
**LST**	**−14.5675**	**4.8026**	**0.002**

LST, land surface temperature; PET, potential evapotranspiration. Bold type indicates statistically significant factor.

### Comparison of reproductive phenology and seed production between CH and CL plants

**Fig. 3.**
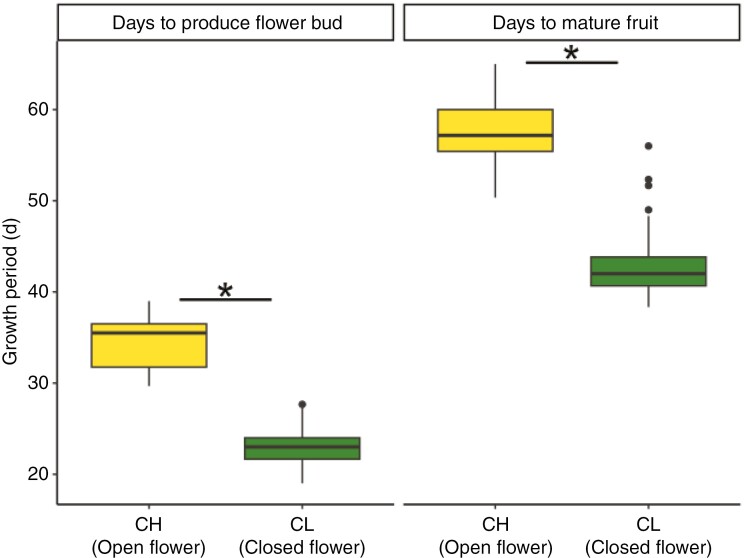
Growth periods of chasmogamous (CH) and cleistogamous (CL) plants. Box plots show the median (horizontal line), 25th and 75th percentiles (bottom and top edges, respectively), and outliers (dots). Asterisk indicates significant (*P* < 0.01) differences between CH and CL individuals on the basis of a linear mixed model.

**Fig. 4.**
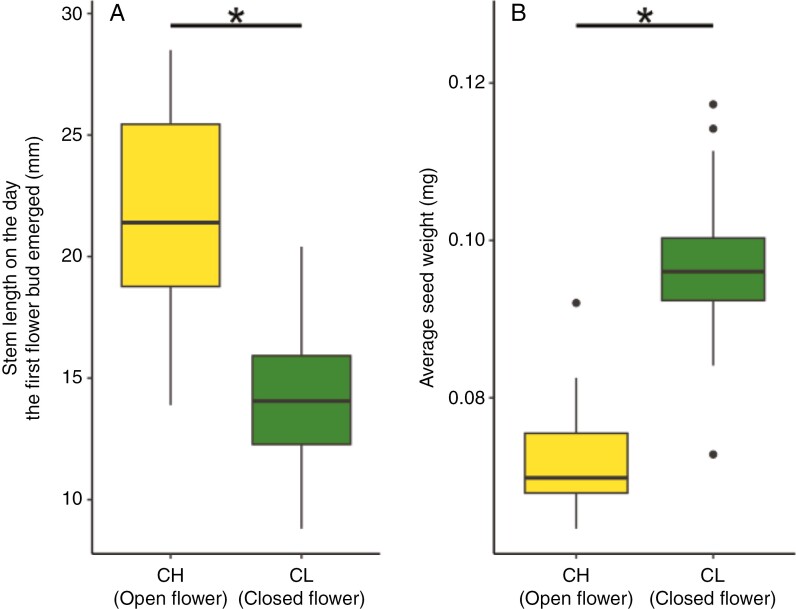
Stem length on the day of emergence of the first flower bud (A) and average seed weight (B) in chasmogamous (CH) and cleistogamous (CL) plants. Box plots show the median (horizontal line), 25th and 75th percentiles (bottom and top edges, respectively), and outlier (dots). Asterisk indicates significant (*P* < 0.01) differences between CH and CL individuals based on a linear mixed model.

We investigated whether the lengths of vegetative growth and seed production differed between CH and CL plants. The onset of flower bud production and fruit maturity occurred significantly earlier in CL plants than in CH plants (LMM, χ^2^ = 363.3 and 247.3, *P* < 0.01 for bud production and fruit maturity, respectively, Fig. 3). The stems were shorter in CL plants than in CH plants on the day of emergence of the first flower bud (LMM, χ^2^ = 85.0, *P* < 0.01, Fig. 4A). Average seed weight was significantly higher in CL plants than in CH plants (LMM, χ^2^ = 135.2, *P* < 0.01, Fig. 4B). Fruit number per individual did not differ between them (Supplementary data Fig. S1a), but the mean number of seeds per fruit was greater in CH plants (LMM, χ^2^ = 115.4, *P* < 0.01, Fig. S1b). Consequently, the estimated number of seeds per individual was significantly higher in CH plants (LMM, χ^2^ = 35.0, *P* < 0.01, Fig. S1c). We compared the lengths of vegetative growth and seed production between CH and CL plants from the rural population (Figs S2–S4). CL plants exhibited earlier phenology (LMM, χ^2^ = 167.4 and 157.8, *P* < 0.01 for bud production and fruit maturity, respectively, Fig. S2) and their seeds were heavier than those of CH plants (LMM, χ^2^ = 91.4, *P* < 0.01, Fig. S3b). We did not conduct this analysis for the urban population because of the small number of CH plants.

### Comparison of reproductive phenology and seed production of CL plants between rural and urban populations

We examined the differences in lengths of vegetative growth and seed production of CL plants between rural and urban populations (Supplementary Data Figs S5–S7); CH plants were excluded because of their limited number. The differences were not significant, but the fruits tended to mature earlier in urban than in rural individuals (LMM, χ^2^ = 1.4, *P* = 0.23 for bud production and χ^2^ = 1.9, *P* = 0.17 for fruit maturity, respectively, Fig. S5).

## DISCUSSION

Our findings indicate that urbanization may drive an evolutionary change in the cleistogamous reproductive system of *P*. *oleracea*. CH plants of *P*. *oleracea* were overall less common than CL plants, and were particularly scarce in urban areas (Fig. 2). During urbanization, CL plants, which are better adapted to the urban environment, may be favourably selected.

CL plants had traits indicative of heat and drought stress avoidance: bolting was initiated and maturity was reached significantly earlier in the CL plants than in the CH plants under the same conditions in our common garden experiment (Fig. 3). Theoretically, an earlier life cycle helps to avoid drought stress and reduces the risk of death before reproduction (Kigel *et al.*, 2011). Rapid evolution towards earlier reproductive phenology under heat and drought conditions has been documented for numerous plant species (Ivey and Carr, 2012; Wolfe and Tonsor, 2014; Sakaguchi *et al.*, 2019; Metz *et al.*, 2020; Blanco-Sánchez *et al.*, 2022; Rauschkolb *et al.*, 2022). Urban areas are renowned for their altered microclimates, in particular higher temperatures than in surrounding rural areas, known as the urban heat island effect (Theeuwes *et al.*, 2017). Urban soils are often compacted and surrounded by a high density of impervious surfaces; they tend to be drier than their rural counterparts (Schmidt *et al.*, 2014). CL plants with earlier reproductive phenology may have an advantage in habitats with a relatively short growth season. These results seem to contradict those of Furukawa *et al*. (2020), who showed that the probability of occurrence of *P*. *oleracea* CL plants increases as mean temperature decreases in their original population. However, it is possible that these findings are limited to certain conditions, such as areas with lower temperatures or cities influenced by the heat island effect, where plants have shorter growing seasons.

It is important to note that plants may exhibit a certain level of plasticity when grown under different conditions. Furukawa *et al.* (2020) cultivated *P*. *oleracea* under high or low temperature and found that all CH plants opened their flowers under high temperature, but the probability of flower opening decreased under low temperature. Nutrient availability (Culley and Klooster, 2007) and disturbance (Carbone *et al.*, 2021) can influence the production of CH or CL flowers in other cleistogamous species. A more thorough analysis is necessary to understand the plasticity in flower production in *P. oleracea*.

CL plants from the urban population tended to ripen earlier than their rural counterparts (Supplementary Data Fig. S5). However, we did not conduct any direct measurements to confirm that early ripening enhances fitness under chronic heat and drought stress. Future studies should incorporate temperature-treatment experiments to ascertain the importance of this trait and elucidate its physiological mechanisms.

The seeds produced on CL plants were significantly heavier than those on CH plants (Fig. 4B), in agreement with studies that have reported a positive correlation between drought conditions and seed size (Leishman and Westoby, 1994; Hallett *et al.*, 2011; Razzaque *et al.*, 2023). For instance, Razzaque *et al.* (2023) observed that *Panicum hallii* seeds were larger in xeric than in mesic ecotypes. Larger seeds potentially enable seedlings to develop more extensive root systems, thus improving water access in arid environments (Leishman and Westoby, 1994; Hallett *et al.*, 2011; Razzaque *et al.*, 2023). Larger seeds may have thicker seed coats, which increase drought tolerance (Lamont and Pausas, 2023). The larger seeds of CL plants may be an adaptive trait in urban environments, where heat and drought stress can be aggravated. However, it is important to interpret these results with caution because all the seeds of CH plants analysed in this study were produced through self-fertilization. Inbred progeny typically have smaller seeds due to the negative effects of inbreeding depression in outcrossing plants (Baldwin and Schoen, 2019; Cheptou, 2019). Therefore, further research is necessary to investigate the characteristics of both seeds and seedlings of *P*. *oleracea*, especially regarding the effects of self-fertilization.

Here, we focused primarily on the evolution of *P*. *oleracea* towards the prevalence of CL plants in urban environments and on the role of abiotic factors. However, there is also room to examine biotic factors. For instance, a decline in the abundance of pollinators can lead to the evolution of traits favourable to self-pollination (Brys and Jacquemyn, 2012; Opedal *et al.*, 2017). In *Centaurium erythraea*, a non-cleistogamous species, the distance between male and female organs decreases in environments with limited pollinators (herkogamy; Brys and Jacquemyn, 2012). Pollinators are a selective force for the number of cleistogamous flowers in *Impatiens capensis* (Panique and Caruso, 2020). While urbanization can influence pollinator species in various ways (Winfree *et al.*, 2007; Potts *et al.*, 2010; Bates *et al.*, 2011), significant declines in pollinators in highly urbanized areas have been reported globally (Hennig and Ghazoul, 2011; Pauw and Louw, 2012; Geslin *et al.*, 2013). Should the decline in pollinators due to urbanization be a direct evolutionary cause for the prevalence of CL plants, the earlier reproductive phenology and larger seeds of CL plants might be evolutionary by-products of adaptation to pollinator scarcity (Fukano *et al.*, 2020). Alternatively, self-fertilization may emerge in rapidly desiccating habitats as a result of selection for drought avoidance, owing to the benefits of accelerated development within flowers (Ivey and Carr, 2012). Future studies should focus on identifying the direct evolutionary drivers behind the shift in *P*. *oleracea* towards CL plants in urban areas.

Genetic differences between urban and rural individuals could also be caused by non-adaptive events such as genetic drift or plant introduction route. We cannot exclude that these neutral events may have contributed to the phenotypic divergence between rural and urban populations of *P*. *oleracea*. Future studies based on broader sampling and molecular genetic analysis should investigate the relative importance of adaptive evolution.

Our results contribute to the growing body of knowledge on plant evolution in urban areas. Environmental features associated with urbanization drive the evolution of leaf traits (Santangelo, 2022*a*; Fukano *et al.*, 2023) and dispersal traits (Cheptou *et al.*, 2008; Palacio and Ordano, 2023). Few studies have examined the evolutionary consequences of urbanization on plant reproductive traits (Brys and Jacquemyn, 2012; Ushimaru *et al.*, 2014). Our study suggests that urbanization can cause rapid evolution of these traits. It is necessary to analyse the evolutionary effects of urbanization on reproductive traits such as flower size (Acoca-Pidolle *et al.*, 2024), floral odour (Petrén *et al.*, 2021) and herkogamy (Brys and Jacquemyn, 2012) in many other taxa. The impact of reproductive traits on the genetic structure of populations also needs to be analysed (Cheptou, 2019). Understanding the above could help understand the potential of plant species to cope with upcoming global changes.

## SUPPLEMENTARY DATA

Supplementary data are available at *Annals of Botany* online and consist of the following.

Figure S1: Number of fruits per individual (a), number of seeds per fruit (b) and number of seeds per individual (c) in chasmogamous (CH) and cleistogamous (CL) plants. Figure S2: Growth periods of chasmogamous (CH) and cleistogamous (CL) individuals in the rural population. Figure S3: Stem length on the day of the emergence of the first flower bud (a) and average seed weight (b) in chasmogamous (CH) and cleistogamous (CL) individuals in the rural population. Figure S4: Number of fruits per individual (a), number of seeds per fruit (b) and number of seeds per individual (c) in chasmogamous (CH) and cleistogamous (CL) individuals from the rural population. Figure S5: Growth periods of cleistogamous (CL) individuals derived from the rural and urban populations. Figure S6: Stem length at the beginning of flower bud emergence (a) and average seed weight (b) in cleistogamous (CL) individuals derived from the rural and urban populations. Figure S7: Fruit counts (a), number of seeds per fruit (b) and number of seeds per individual (c) in cleistogamous (CL) individuals derived from the rural and urban populations. Table S1. List of the seed sampling points. Table S2. List of environmental variables around the seed sampling locations.

mcae105_suppl_Supplementary_Material
