# Supplementary material for: The flower does not open in the city: evolution of plant reproductive traits of Portulaca oleracea in urban populations
Source: Ann Bot. 2024 Aug 1;135(1-2):269–76. doi: 10.1093/aob/mcae105 (PMC11805939; doi:10.1093/aob/mcae105)
Supplement: mcae105_suppl_Supplementary_Material [file mcae105_suppl_supplementary_material.docx]

Supplementary data Figure S1

Number of fruits per individual (a), number of seeds per fruit (b), and number of seeds per individual (c) in chasmogamous (CH) and cleistogamous (CL) plants. Box plots show the median (horizontal line), 25th and 75th percentiles (bottom and top edges, respectively), and outliers (dots). Asterisks indicate significant (*p* < 0.01) differences between CH and CL individuals on the basis of a Linear Mixed Model.


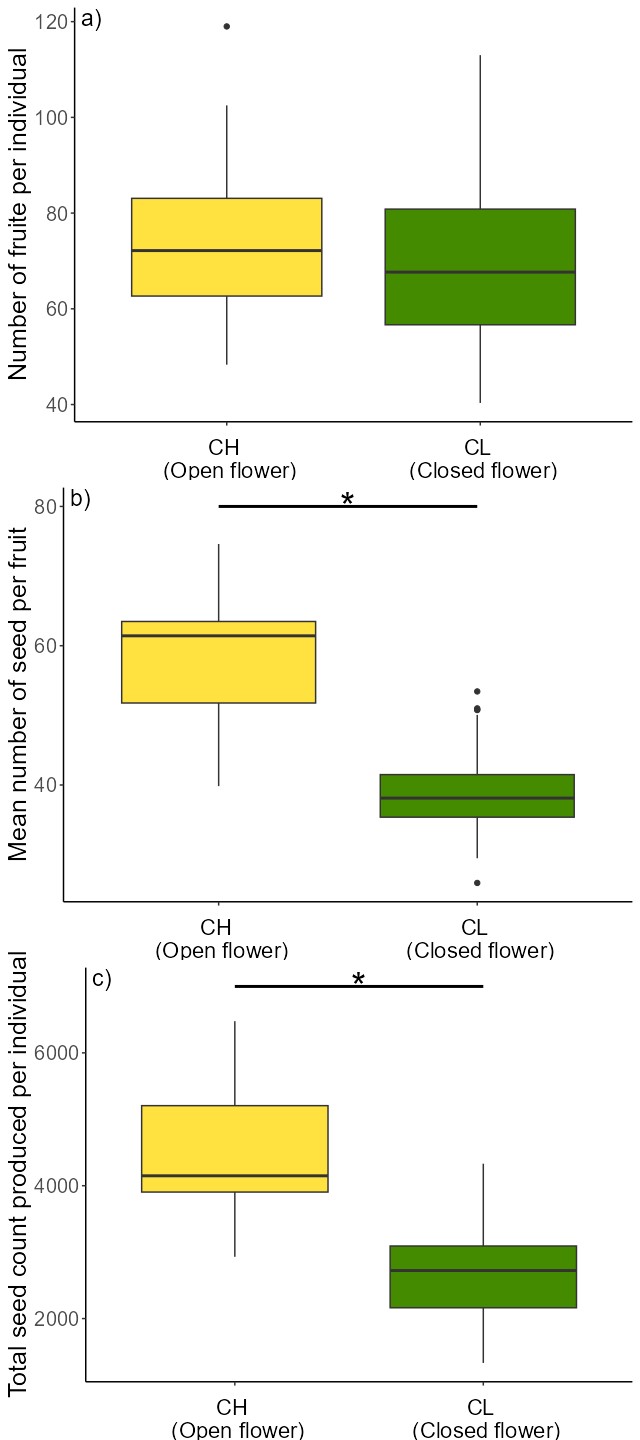


Supplementary data Figure S2

Growth periods of chasmogamous (CH) and cleistogamous (CL) individuals in the rural population. Box plots show the median (horizontal line), 25th and 75th percentiles (bottom and top edges, respectively), and outliers (dots). Asterisks indicate significant (*p* < 0.01) differences between CH and CL individuals on the basis of a Linear Mixed Model.


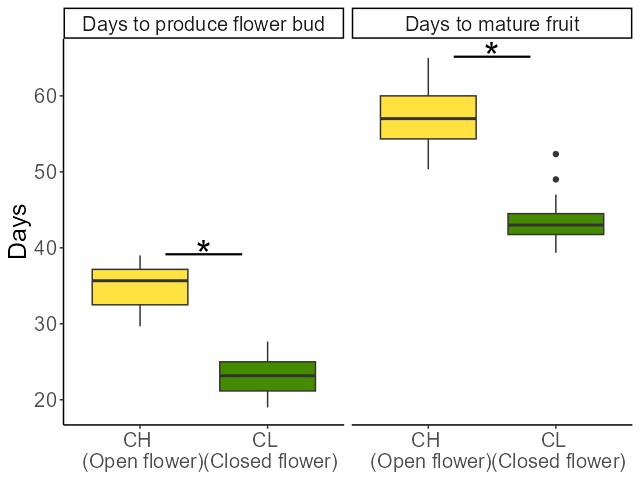


Supplementary data Figure S3

Stem length on the day of the emergence of the first flower bud (a) and average seed weight (b) in chasmogamous (CH) and cleistogamous (CL) individuals in the rural population. Box plots show the median (horizontal line), 25th and 75th percentiles (bottom and top edges, respectively), and an outlier (dot). Asterisks indicate significant (*p* < 0.01) differences between CH and CL individuals on the basis of a Linear Mixed Model.


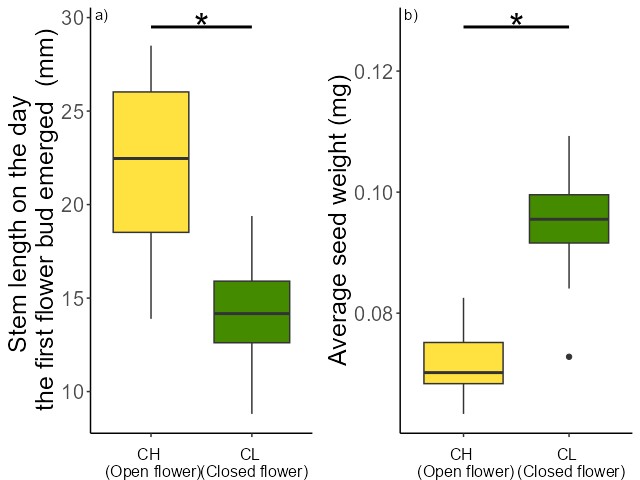


Supplementary data Figure S4

Number of fruits per individual (a), number of seeds per fruit (b), and number of seeds per individual (c) in chasmogamous (CH) and cleistogamous (CL) individuals from the rural population. Box plots show the median (horizontal line), 25th and 75th percentiles (bottom and top edges, respectively), and an outlier (dot). Asterisks indicate significant (*p* < 0.01) differences between CH and CL individuals on the basis of a Linear Mixed Model.


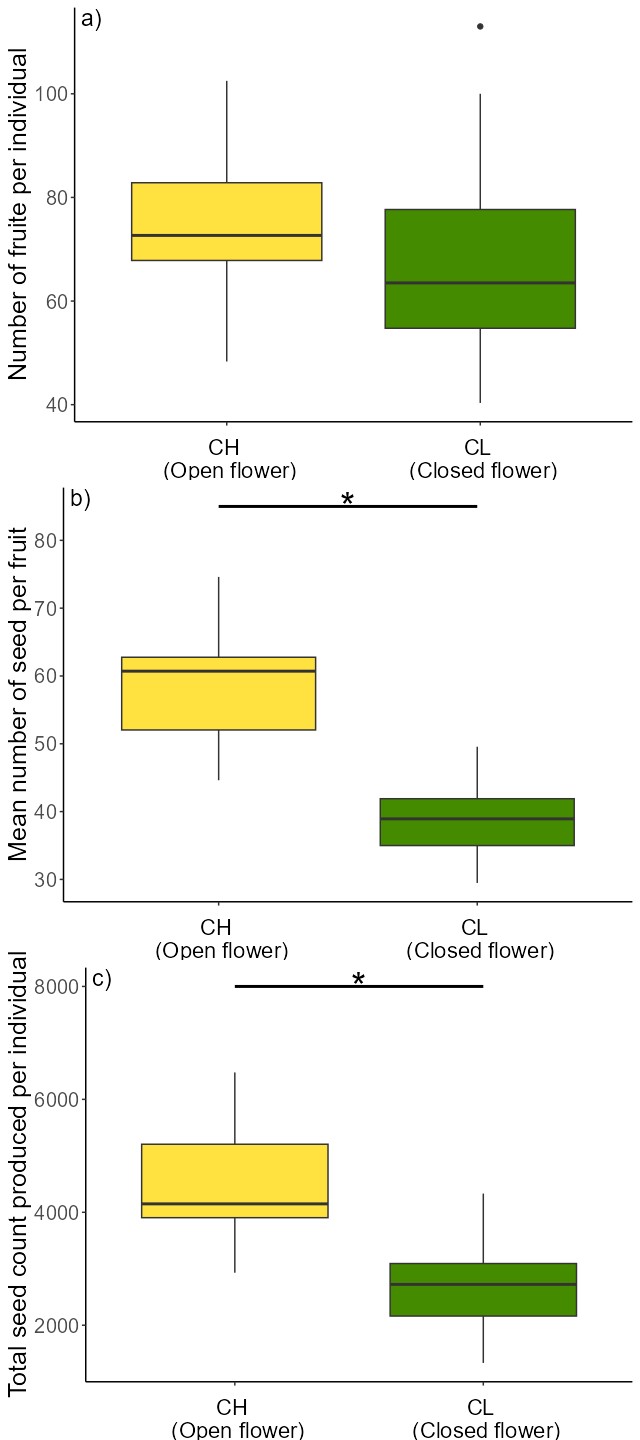


Supplementary data Figure S5

Growth periods of cleistogamous (CL) individuals derived from the rural and urban populations. Box plots show the median (horizontal line), 25th and 75th percentiles (bottom and top edges, respectively), and outliers (dots).


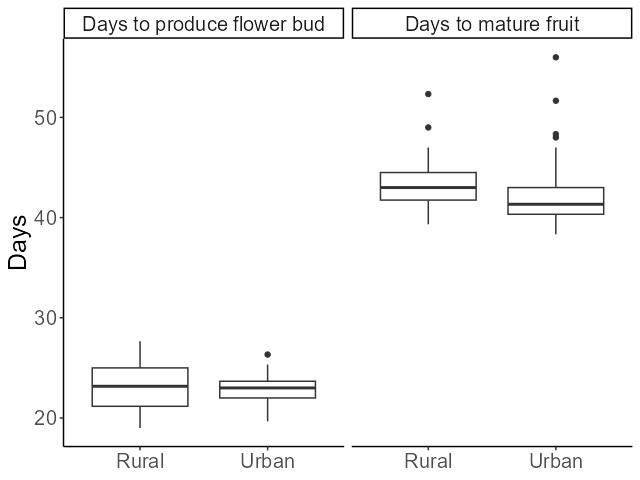


Supplementary data Figure S6

Stem length at the beginning of flower bud emergence (a) and average seed weight (b) in cleistogamous (CL) individuals derived from the rural and urban populations. Box plots show the median (horizontal line), 25th and 75th percentiles (bottom and top edges, respectively), and outliers (dots).


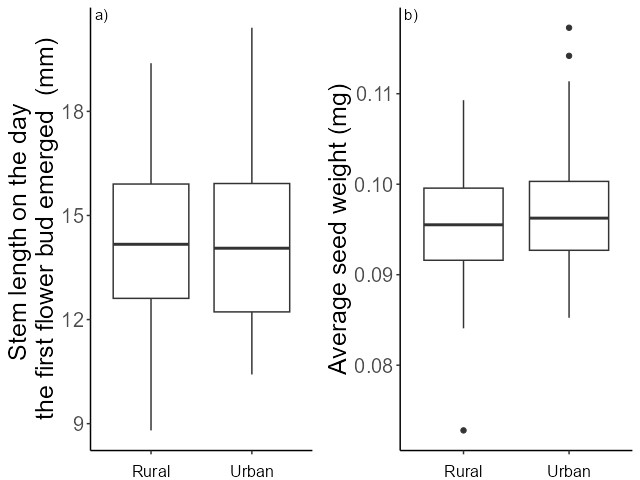


Supplementary data Figure S7

Fruit counts (a), number of seeds per fruit (b), and number of seeds per individual (c) in cleistogamous (CL) individuals derived from the rural and urban populations. Box plots show the median (horizontal line), 25th and 75th percentiles (bottom and top edges, respectively), and outliers (dots).


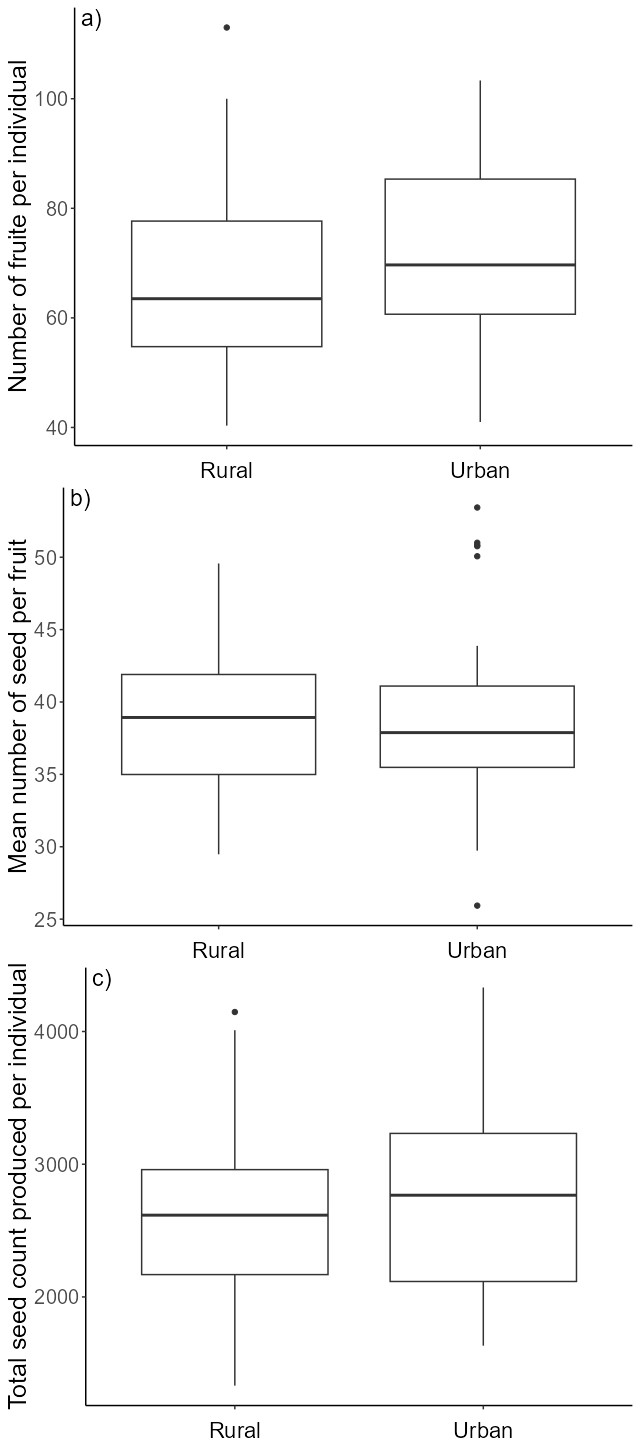


Supplementary data Table S1. List of the seed sampling points.

| **No.** | **Type of habitat** | **Prefecture** | **Longitude** | **Latitude** | **No. of F1 generation** | **No. of F2 generation** |
| --- | --- | --- | --- | --- | --- | --- |
| R-1 | Rural | Tokyo | 35.714 | 139.313 | 2 | 2 |
| R-2 | Rural | Ibaraki | 35.996 | 140.195 | 5 | 5 |
| R-3 | Rural | Ibaraki | 36.268 | 140.256 | 5 | 5 |
| R-4 | Rural | Ibaraki | 36.231 | 140.159 | 6 | 5 |
| R-5 | Rural | Tochigi | 36.301 | 139.632 | 5 | 5 |
| R-6 | Rural | Ibaraki | 36.188 | 140.022 | 5 | 5 |
| R-7 | Rural | Ibaraki | 36.154 | 140.122 | 6 | 6 |
| R-8 | Rural | Saitama | 35.977 | 139.333 | 7 | 7 |
| R-9 | Rural | Ibaraki | 36.141 | 139.850 | 6 | 5 |
| R-10 | Rural | Saitama | 36.078 | 139.344 | 2 | 0 |
| U-1 | Urban | Tokyo | 35.730 | 139.704 | 8 | 6 |
| U-2 | Urban | Tokyo | 35.711 | 139.821 | 8 | 8 |
| U-3 | Urban | Tokyo | 35.689 | 139.773 | 3 | 3 |
| U-4 | Urban | Tokyo | 35.606 | 139.692 | 5 | 5 |
| U-5 | Urban | Tokyo | 35.634 | 139.735 | 5 | 4 |
| U-6 | Urban | Tokyo | 35.656 | 139.684 | 7 | 7 |
| U-7 | Urban | Tokyo | 35.700 | 139.695 | 2 | 1 |
| U-8 | Urban | Tokyo | 35.735 | 139.738 | 3 | 3 |
| U-9 | Urban | Tokyo | 35.727 | 139.766 | 6 | 6 |
| U-10 | Urban | Tokyo | 35.633 | 139.702 | 9 | 9 |

Supplementary data Table S2. List of environmental variables around the seed sampling locations

| **Variable** | **Time of data collection** | **Resolution** | **Source** |
| --- | --- | --- | --- |
| Land surface temperature (LST) | Average 2000–2008 | 100 m | ASTER GED (https://lpdaac.usgs.gov/products/ag100v003/) |
| Summer vegetation cover quantified using the normalized vegetation index (NDVI) | 2012 (Jul.–Sep. average) | 250 m | GSI (https://www.gsi.go.jp/kankyochiri/ndvi-Modis_download.html) |
| Elevation assessed using a digital elevation model | 2009 | 10 m | MLIT of Japan (https://nlftp.mlit.go.jp/ksj/) |
| Annual aridity index (AI) | Average 1970–2000 | 1 km | Global-AI_PET_v3 (https://csidotinfo.wordpress.com/2019/01/24/global-aridity-index-and-potential-evapotranspiration-climate-database-v3/) |
| Potential evapotranspiration (PET) | Average 1970–2000 | 1 km | Global-AI_PET_v3 (https://csidotinfo.wordpress.com/2019/01/24/global-aridity-index-and-potential-evapotranspiration-climate-database-v3/) |
| Global man-made impervious surface | 2010 | 30 m | SEDAC (https://sedac.ciesin.columbia.edu/data/set/ulandsat-gmis-v1/data-download) |
